# Avaliação de Padrões Pressóricos Dipper e Não-Dipper e Qualidade de Vida entre Pacientes com Doença Pulmonar Obstrutiva Crônica

**DOI:** 10.36660/abc.20190536

**Published:** 2021-01-13

**Authors:** Meryem Askın, Esra Meltem Koc, Kaan Sozmen, Muzaffer Onur Turan, Zeki Soypacacı, Saliha Aksun

**Affiliations:** 1 Izmir Katip Celebi University Faculty of Medicine Department of Family Medicine Izmir Turquia Izmir Katip Celebi University Faculty of Medicine - Department of Family Medicine, Izmir - Turquia; 2 Izmir Katip Celebi University Faculty of Medicine Department of Public Health Izmir Turquia Izmir Katip Celebi University Faculty of Medicine - Department of Public Health, Izmir - Turquia; 3 Izmir Katip Celebi University Faculty of Medicine Department of Chest Diseases Izmir Turquia Izmir Katip Celebi University Faculty of Medicine - Department of Chest Diseases,Izmir - Turquia; 4 Izmir Katip Celebi University Faculty of Medicine Department of Nephrology Izmir Turquia Izmir Katip Celebi University Faculty of Medicine - Department of Nephrology, Izmir - Turquia; 5 Izmir Katip Celebi University Faculty of Medicine Department of Medical Biochemistry Izmir Turquia Izmir Katip Celebi University Faculty of Medicine - Department of Medical Biochemistry, Izmir - Turquia

**Keywords:** Doença Pulmonar Obstrutiva Crônica, Doenças Cardiovasculares, Monitoramento, Dipper, Não Dipper, Prognóstico, Qualidade de Vida

## Abstract

**Fundamento:**

O padrão pressórico não-dipper é definido por uma redução inferior a 10% na pressão arterial noturna e está associado a doenças cardiovasculares. Acredita-se que a inflamação desempenhe um papel na patogênese da doença pulmonar obstrutiva crônica (DPOC) e no padrão pressórico não-dipper e ambas as doenças estão associadas a uma qualidade de vida mais baixa.

**Objetivo:**

O objetivo deste estudo foi o de investigar os efeitos do padrão pressórico não-dipper em pacientes com DPOC.

**Métodos:**

Foi realizado um estudo transversal incluindo 142 pacientes com DPOC. O Questionário Respiratório de Saint George e a Escala de Qualidade de Vida Euro foram utilizados para a coleta de dados. Para entender a rigidez arterial, o índice de aumento e a velocidade da onda de pulso foram medidos; subsequentemente, foi realizada a monitorização ambulatorial da pressão arterial de 24 horas. Foi aplicado um modelo de regressão logística multivariável para entender a relação entre as diferentes variáveis independentes e o padrão pressórico. Foram considerados estatisticamente significativos valores de p inferiores a 0,05.

**Resultados:**

Como resultado, 76,1% (n = 108) dos pacientes apresentaram o padrão pressórico não-dipper. Os pacientes com padrão não-dipper apresentaram valores mais altos de proteína C reativa (OR: 1,123; IC 95%: 1,016;1,242), índice de aumento (OR: 1,057; IC 95%: 1,011;1,105) e pontuação total no Questionário Respiratório de Saint George (OR: 1,021; IC 95%: 1,001;1,042), em comparação com os pacientes com padrão dipper. Adicionalmente, com o aumento do número de pessoas que habitavam o domicílio, verificou-se que o padrão pressórico não-dipper era mais frequente (OR: 1,339; IC 95%:1,009;1,777).

**Conclusão:**

O padrão pressórico não-dipper pode aumentar o risco cardiovascular ao desencadear a inflamação e pode afetar adversamente o prognóstico da DPOC diminuindo a qualidade de vida relacionada à doença. (Arq Bras Cardiol. 2020; [online].ahead print, PP.0-0)

## Introdução

A doença pulmonar obstrutiva crônica (DPOC) é altamente prevalente em todo o mundo; caracteriza-se pela limitação do fluxo aéreo e, segundo a Organização Mundial da Saúde, será a terceira causa de morte em 2030.^1^

Uma redução de mais de 10% da pressão arterial noturna é um processo fisiológico^2^. Quando a redução da pressão arterial noturna é inferior a 10%, isto é chamado de padrão pressórico não-dipper, que é conhecido por estar associado a doenças cardiovasculares e lesões em órgãos-alvo.^2^ O índice de aumento (Aix) e a velocidade da onda de pulso (VOP) são indicadores de rigidez arterial que podem prever eventos cardiovasculares futuros, e os valores de Aix e VOP são mais elevados no padrão pressórico não-dipper.^3 , 4^ É controverso como o padrão pressórico não-dipper causa esses efeitos, mas acredita-se que esse padrão induz a expressão de citocinas do endotélio e desencadeia um processo inflamatório.^5^ Os marcadores de inflamação, como a proteína C reativa (PCR), a relação neutrófilos/linfócitos (NLR) e a relação plaquetas/linfócitos (PLR), são mais altos entre os indivíduos com pressão arterial não-dipper em comparação com seus pares.^5^

A inflamação sistêmica desempenha um papel importante na patogênese da DPOC, e acredita-se que a PCR e a NLR podem ser usados como marcadores diagnósticos e prognósticos da DPOC.^6 , 7^ A presença de um processo inflamatório na patogênese tanto da DPOC quanto da pressão arterial não-dipper sugere que a incidência de padrão pressórico não-dipper possa ser alta em pacientes DPOC.^8^ Seus efeitos negativos na qualidade de vida constituem outro ponto comum de ambas as doenças.^9 , 10^ A qualidade de vida é a percepção de saúde do indivíduo e tem um impacto direto na sua saúde física e mental; também está relacionado ao número de exacerbações e hospitalizações agudas na DPOC.^9^

Estudos que investigaram os efeitos dos padrões de pressão arterial na DPOC são bastante limitados e a elucidação deste assunto pode ser um guia para o manejo da DPOC. O objetivo deste estudo é investigar o efeito do padrão pressórico não-dipper nos valores laboratoriais e na qualidade de vida na DPOC.

## Métodos

O diagnóstico da DPOC deve ser confirmado por espirometria em pacientes com dispneia, tosse crônica, produção crônica de expectoração e exposição a fatores de risco de DPOC. Em nosso estudo, pacientes com diagnóstico de DPOC por especialistas em doença torácica e, portanto, em uso de medicação, foram aceitos como DPOC. Além disso, os exames espirométricos dos pacientes foram verificados pelo sistema de computador.

### Participantes

Este estudo transversal foi realizado em pacientes com DPOC, com idades entre 18 e 80 anos, que foram admitidos à clínica de internamento ou ambulatória para doenças pulmonares do Hospital de Treinamento e Pesquisa Atatürk da Universidade Izmir Katip Celebi. O estudo excluiu pacientes que, além da DPOC, apresentavam infecção ativa conhecida, malignidade, insuficiência cardíaca congestiva, diabetes mellitus ou insuficiência renal. O estudo foi realizado entre janeiro e junho de 2018.

### Tamanho da amostra

O tamanho da amostra foi calculado por meio do programa GPOWER 3.1. Usando o achado de uma pesquisa anterior, presumimos que os níveis médios de PCR seriam de 4,9 ± 1,7 mg/lt no grupo não-dipper e de 3,8 ± 1,5 mg/lt no grupo dipper. Para mostrar as diferenças entre os dois grupos, o tamanho da amostra necessário foi calculado em 128, com poder de 95% e uma taxa de erro tipo 1 bilateral de 5%. Aumentamos o tamanho da amostra em 10% e visamos chegar a 140 pacientes.^11^

### Ferramentas de coleta de dados

#### Características sociodemográficas

Para examinar os dados sociodemográficos, o nível de escolaridade foi categorizado em dois grupos (ensino fundamental 2 ou básico e ensino médio ou superior). A faixa de renda foi categorizado em três grupos (≥ 1.500 liras turcas (TL), 1.501 a 3.499 TL e ≤ 3.500 TL). Os valores do índice de massa corporal foram divididos em três categorias (normal [≤ 24,99 kg/m^2^], sobrepeso [25 a 29,99 kg/m^2^] e obeso [≥ 30 kg/m^2^]). O estado civil foi dividido em dois grupos (solteiro e casado). O consumo de álcool também foi dividido em dois grupos (sim e não). Os locais onde os pacientes foram incluídos no estudo foram divididos em dois grupos (serviço de internação e ambulatório) e o histórico familiar de DPOC foi categorizado em dois grupos (sim e não). Os pacientes com diagnóstico de hipertensão por um médico e em uso de medicamentos prescritos foram considerados hipertensos. Pacientes que fumavam regularmente, pelo menos uma vez ao dia, foram considerados fumantes ativos. Em relação às variáveis contínuas, os pacientes foram solicitados a responder a perguntas como idade, número de pessoas que moravam em seus domicílios, anos de tabagismo e número de maços, número de admissões na unidade de emergência devido à DPOC durante o último ano e número de internações hospitalares por DPOC.

#### Questionário Respiratório de Saint George

O Questionário Respiratório de Saint George (SGRQ) é uma escala de qualidade de vida desenvolvida especialmente para o sistema respiratório que pode ser utilizada em pacientes com asma e DPOC, bem como em pacientes com bronquiectasia e sarcoidose.^12^ A escala inclui os componentes de sintomas, atividade e efeito; no final, são obtidas as pontuações de todos os três componentes e da qualidade de vida total.^12^ A escala é pontuada de 0 a 100. Para cada componente e para a pontuação total produzida, ‘0’ indica ‘perfeito’ e ‘100’ indica a ‘pior’ qualidade de vida.^12^ A validade e a confiabilidade da versão turca da escala foram confirmadas.^12^

#### Escala de Qualidade de Vida Euro

A Escala de Qualidade de Vida Euro (EQ-5D) é uma escala de qualidade de vida geral de 5 questões, cada uma consistindo de 3 níveis.^13^ A escala é composta por 5 dimensões, incluindo mobilidade, autocuidado, atividades diárias, dor e humor. As pontuações mais altas indicam maior qualidade de vida.^13^ A utilidade da pontuação EQ-5D foi calculada usando o algoritmo MVH-A1 por Dolan.^14^ Este algoritmo fornece uma faixa de −0,594 a +1, onde valores mais altos indicam melhor qualidade de vida. A validade e a confiabilidade da escala foram confirmadas para o contexto turco.^13^

#### Índice de aumento e velocidade da onda de pulso

Após a aplicação do questionário e da coleta de sangue, os pacientes foram examinados quanto ao Aix e à VOP. Um aparelho Mobil-O-Graph® (IEM; Stolberg, Alemanha) calculou o Aix e a VOP registrando a pressão arterial braquial oscilométrica; o manguito subsequentemente reinsuflou na fase diastólica durante aproximadamente 10 segundos, registrando as ondas de pulso braquial com um sensor de pressão de alta fidelidade.

#### Monitorização ambulatorial da pressão arterial de 24 horas e coleta de sangue

Para medição da pressão arterial de 24 horas, os pacientes foram monitorados com um dispositivo Mobil-O-Graph NG® (IEM; Stolberg, Alemanha). O dispositivo foi ajustado para realizar medição de 24 horas, uma vez a cada 15 minutos durante o dia e uma vez a cada 30 minutos à noite. No dia seguinte, no mesmo horário, os pacientes devolveram os dispositivos. O braço não dominante foi usado para a medição, e as medidas diurnas e noturnas foram ajustadas de acordo com as horas de sono e vigília dos pacientes. Os pacientes foram orientados a continuar suas atividades habituais e a evitar exercícios exaustivos. Os exames de sangue foram obtidos dos pacientes antes da administração do tratamento. Entre os pacientes hospitalizados no serviço de doenças pulmonares, o questionário foi aplicado no mesmo dia. O dispositivo de monitorização ambulatorial da pressão arterial de 24 horas (MAPA 24h) foi aplicado em condições semelhantes para os pacientes ambulatoriais.

Os resultados da medição da MAPA 24h foram examinados e, quando os valores médios noturnos das pressões arteriais sistólica e diastólica apresentavam um descenso de 10% ou mais em relação aos valores médios diurnos, considerava-se como pressão arterial dipper.^2^ Quando o descenso da pressão arterial foi inferior a 10%, foi considerada como padrão pressórico não dipper^2^. Os exames de sangue de PCR foram realizados no analisador automático Architect C16000 (Abbott Diag., EUA) e o hemograma foi analisado no dispositivo de sangue total Mindray BC-6800 (Mindray, China).

#### Aprovação ética

A aprovação ética deste estudo foi obtida do Comitê de Ética em Pesquisa Clínica Intervencionista da Universidade Izmir Katip Celebi sob parecer número 164 (data de aprovação: 22 de dezembro de 2016). Todos os procedimentos deste estudo estavam de acordo com a Declaração de Helsinque de 1975, atualizada em 2013. Foi obtido o consentimento informado de todos os participantes incluídos no estudo.

## Análise estatística

A análise estatística foi realizada no SPSS versão 16. Neste estudo, a distribuição das variáveis contínuas foi avaliada pelo teste de Kolmogorov-Smirnov e os dados não foram distribuídos normalmente. As variáveis contínuas foram apresentadas como medianas e intervalos interquartis (IIQ). A comparação estatística dos dois grupos independentes foi realizada usando o teste U de Mann Whitney. As comparações entre grupos de variáveis categóricas foram realizadas por meio do teste do qui-quadrado. A força de associação entre duas variáveis contínuas foi avaliada por meio de testes de correlação de Spearman.

Os efeitos independentes com respeito à presença de pressão arterial não-dipper dos diferentes fatores de identificação foram examinados usando modelos de regressão logística. O teste de Hosmer-Lemeshow foi usado para avaliar o ajuste do modelo. As variáveis independentes com p ≤ 0,10 na análise bivariada foram incluídas no modelo de regressão logística multivariada com método de eliminação para trás. Devido à alta correlação entre a pressão sanguínea sistólica noturna e a pressão arterial média noturna, apenas a pressão arterial média noturna foi adicionada ao modelo. Os coeficientes ajustados são apresentados com seus intervalos de confiança de 95%. Todos os valores de p foram bicaudais e os valores de p inferiores a 0,05 foram considerados estatisticamente significativos.

## Resultados

Foram convidados para o estudo 167 pacientes; destes, 12 não quiseram usar o aparelho MAPA 24h ou não quiseram participar no estudo devido a falta de tempo. Foram excluídos 13 pacientes devido a medidas inadequadas de MAPA 24h, e o estudo foi concluído com 142 pacientes. No total, 23,9% (n = 34) dos pacientes tinham padrão pressórico dipper e 76,1% (n = 108) tinham padrão não dipper. Desses pacientes, 43% (n = 61) foram incluídos da clínica de internação e 57% (n = 81) eram pacientes ambulatoriais.

De acordo com a análise univariada, as variáveis sociodemográficas como idade, sexo e estado civil não afetaram significativamente o padrão pressórico. Por outro lado, o número mediano de pessoas que habitavam o domicílio foi mais alto nos pacientes que tinham padrão pressórico não-dipper em comparação com aqueles com padrão pressórico dipper. A relação entre as variáveis sociodemográficas e o padrão pressórico é apresentada na Tabela 1 .

**Tabela 1:** – Variáveis sócio-demográficas

Variáveis categóricas	Dipper	Não-dipper	Total	Valor p
	**(n = 34)**	**(n = 108)**	**(n = 142)**	
**Sexo n (%)**				
Feminino	7 (20,6)	25(23,1)	32 (22,5)	0,819
Masculino	27 (79,4)	83 (76,9)	110 (77,5)	
**Índice de massa corporal n (%)**				
Normal (18,5 a 24,5)	19 (55,9)	51 (47,2)	70 (49,3)	0,571
Sobrepeso (25 a 29,9)	10 (29,4)	33 (30,6)	43 (30,3)	
Obeso (30 a 40)	5 (14,7)	24 (22,2)	29 (20,4)	
**Nível de educação n (%)**				
Ensino fundamental 2 ou básico	11 (32,4)	43 (39,8)	54(38)	0,544
Ensino médio ou superior	23(67,6)	65 (60,2)	88 (62)	
**Faixa de renda n (%)**				
1500 liras turcas ou menos (baixa)	0 (0)	9 (8,3)	9 (6,3)	0,217
1501 a 3500 liras turcas (média)	20 (58,8)	60 (55,6)	80 (56,3)	
3501 liras turcas ou mais (alta)	14 (41,2)	39 (36,1)	53 (37,3)	
**Estado civil n (%)**				
Solteiro	9 (26,5)	24 (22,2)	33 (23,2)	0,644
Casado	25 (73,5)	84 (77,8)	109 (76,8)	
**Lugar de atendimento n (%)**				
Serviço de internamento	11 (32,4)	50 (46,3)	61 (43)	0,169
Clínica ambulatória	23 (67,6)	58 (53,7)	81 (57)	
**Presença de hipertensão n (%)**				
Sim	18 (52,9)	49 (47,1)	67 (48,6)	0,693
Não	16 (47,1)	55 (52,9)	71 (51,4)	
**Histórico familiar de DPOC n (%)**				
Sim	9 (26,5)	43 (39,8)	52 (36,6)	0,221
Não	25 (73,5)	65 (60,2)	90 (63,4)	
**Tabagismo n (%)**				
Sim	8 (23,5)	21 (29,4)	29 (20,4)	0,629
Não	26 (76,5)	87 (80,6)	113 (79,6)	
**Uso de álcool n (%)**				
Sim	12 (35,3)	25 (23,1)	37 (26,1)	0,182
Não	22 (64,7)	83 (76,9)	105 (73,9)	
**Variáveis não categóricas**				
Idade, mediano (IIQ)	65 (59,75-73)	66 (55-71,75)	66 (55,75-72)	0,739
Número de moradores no domicílio, mediano (IIQ)	2 (1-2)	2 (2-3)	2 (2-3)	0,038^*^
Tabagismo, maços-ano, mediano (IIQ)	42,5 (23,75-60)	50 (30-80)	50 (30-74,25)	0,317
Número de visitas ao departamento de emergência, mediano (IIQ)	3,5 (1-6)	3 (1,25-6)	3 (1-6)	0,929
Número de hospitalizações durante o último ano, mediano (IIQ)	2 (0-3)	1 (0-2)	1 (0-2)	0,296

p: percentis; IIQ: intervalo interquartil. * Significância estatística (p < 0,05).

Os valores médios da pressão sanguínea sistólica noturna, pressão arterial média noturna, Aix e PCR foram significativamente maiores entre os participantes com padrão pressórico não-dipper em comparação com seus pares. A associação entre a pressão arterial de 24 horas e os resultados laboratoriais é mostrada na Tabela 2 . Quando a qualidade de vida do paciente foi examinada de acordo com o padrão pressórico, os sintomas, efeitos e pontuações totais do SGRQ foram maiores entre os pacientes com pressão arterial não-dipper. e a pontuação do EQ-5D foi menor; as diferenças foram estatisticamente significativas. ( Tabela 3 ).

**Tabela 2:** – Valores de pressão arterial ambulatorial de 24 horas e resultados laboratoriais

Valores de pressão arterial e resultados laboratoriais	Dipper (n = 34)	Não-dipper (n = 108)	Total (n = 142)	Valor p
PAS média de 24 h, mmHg, mediano (IIQ)	122 (111-129)	122,5 (116-129,75)	122 (115,75-129,25)	0,985
PAD média de 24 h, mmHg, mediano (IIQ)	76 (71- 85,75)	73,5 (67,25-82)	74 (68-84)	0,236
PA média de 24 h, mmHg, mediano (IIQ)	97 (88-113)	97 (90-105)	97 (88,75-106)	0,305
Frequência cardíaca média de 24 h, mediano (IIQ)	84 (69,75-88)	77 (70-84,75)	77,5 (70-88)	0,152
Pressão de pulso de 24 h, mmHg, mediano (IIQ)	47 (37-51)	47 (42-52)	47 (42-51,25)	0,979
PAS média diurna, mmHg, mediano (IIQ)	128 (110,75-145)	124 (116-131)	124,5 (116-133)	0,224
PAD média diurna, mmHg, mediano (IIQ)	74 (71-83)	75 (67-82)	74 (69-82)	0,622
PA média diurna, mmHg, mediano (IIQ)	99 (73,25-91,5)	97 (90-104)	98 (91-106)	0,098
Frequência cardíaca média diurna, mediano (IIQ)	84 (73,25-91,5)	78 (71-86,75)	80 (71-87)	0,127
Pressão de pulso diurna, mmHg, mediano (IIQ)	47 (38-52,25)	47 (42-52)	47 (42-52)	0,781
PAS média noturna, mmHg, mediano (IIQ)	118 (107,75-120)	120 (115-133)	118 (114-131,5)	0,012^*^
PAD média noturna, mmHg, mediano (IIQ)	71 (64,75-73)	72 (65-80)	72 (65-78)	0,099
PA média noturna, mmHg, mediano (IIQ)	94 (82,5-94)	94 (87-104,5)	94 (86-101)	0,045^*^
Frequência cardíaca média noturna, mediano (IIQ)	74,5 (54-88)	73 (61-82)	73 (61-82)	0,698
Pressão de pulso noturna, mmHg, mediano (IIQ)	44 (43,25-51,5)	47,5 (44-52)	47 (44-52)	0,105
Índice de aumento, mediano (IIQ)	20,40 (14,75-24,3)	27 (18-35)	23,05 (17-33)	0,020^*^
Velocidade da onda de pulso, mediano (IIQ)	9,65 (8,67-10,32)	9,2 (8-10,2)	9,45 (8,2-10,8)	0,794
Contagem de leucócitos, mediano (IIQ)	8,85 (7,71-10,63)	9,61 (8,64-11,18)	9,44 (8,32-11,14)	0,073
PCR, mediano (IIQ)	0,97 (0,02-3)	4,34 (1,12-7,42)	2,86 (0,6-6,54)	<0,001^*^
NLR, mediano (IIQ)	2,89 (2,29-5,47)	3,93 (2,28-6,19)	3,76 (2,31-5,88)	0,210
PLR, mediano (IIQ)	130,5 (95,16-203,58)	160,37 (115,38-201,61)	152,06 (107,74-201)	0,110

IIQ: intervalo interquartil; NLR: relação neutrófilos/linfócitos; PA: pressão arterial; PAD: pressão arterial diastólica; PAS: pressão arterial sistólica; PCR: proteína C reativa; PLR: relação plaquetas/linfócitos. * Significância estatística (p < 0,05)

**Tabela 3:** – Pontuações no Questionário Respiratório de Saint George e na Escala de Qualidade de Vida Euro

Pontuações	Dipper (n = 34)	Não-dipper (n =108)	Total (n = 142)	Valor p
SGRQ sintomas, mediano (IIQ)	74,13 (56,43-78,97)	78,08 (64,97-83,68)	76,08 (61,4-82,27)	0,021^*^
SGRQ atividade, mediano (IIQ)	62,49 (48,38-86,72)	74,48 (60,67-87,53)	71,4 (53,83-87,53)	0,091
SGRQ impacto, mediano (IIQ)	47,64 (25,15-67,32)	58,1 (46,33-74,77)	56,99 (38,33-74,3)	0,025^*^
SGRQ total, mediano (IIQ)	53,84 (35,64-73,64)	65,23 (53,84-79,85)	63,71 (52,01-78,63)	0,021^*^
EQ5D, mediano (IIQ)	0,51 (0,51-0,58)	0,51 (0,51-0,51)	0,51 (0,51-0,51)	0,034^*^

EQ5D: Escala de Qualidade de Vida Euro; IIQ: intervalo interquartil; p: percentis; SGRQ: Questionário Respiratório de Saint George. * Significância estatística (p < 0,05)

De acordo com a análise de regressão logística multivariada, a PCR (OR: 1,123; IC 95%: 1,016;1,242), o Aix (OR: 1,057; IC 95%: 1,011;1,105) e a pontuação total do SGRQ (OR: 1.021; IC 95%: 1,001;1,042) eram mais altos nos pacientes com padrão pressórico não-dipper do que naqueles com o padrão dipper. Além disso, os indivíduos com padrão pressórico não-dipper viviam em domicílios mais lotados, em comparação com os indivíduos com padrão pressórico dipper (OR: 1,339; IC 95%: 1,009;1,777) ( Tabela 4 ).

**Tabela 4:** – Análise de regressão logística do padrão pressórico não-dipper

Fatores determinantes	Razão de chances (Univariado)			Razão de chances (Multivariado)		
	β	IC 95%	Valor p	β	IC 95%	Valor p
Idade	0,997	0,961;1,034	0,879	0,995	0,950;1,042	0,817
**Sexo**						
Feminino	1	1	1	1	1	1
Masculino	0,861	0,335;2,212	0,756	1,100	0,341;3,546	0,873
Número de moradores no domicílio	1,169	0,903;1,515	0,236	1,339	1,009;1,777	0,043*
PA média noturna, mmHg	1,038	0,999;1,078	0,055	1,033	0,991;1,077	0,122
Índice de aumento	1,049	1,008;1,092	0,018*	1,057	1,011;1,105	0,015**
PCR	1,141	1,025;1,270	0,016*	1,123	1,016;1,242	0,024*
SGRQ Total	1,023	1,003;1,043	0,025*	1,021	1,001;1,042	0,040*
EQ5D	0,440	0,076;2,540	0,358	0,766	0,118;4,960	0,780

β: coeficiente de regressão; EQ5D: Escala de Qualidade de Vida Euro; IC 95%: intervalo de confiança de 95%; PA: pressão arterial; PCR: proteína C reativa; SGRQ: Questionário Respiratório de Saint George. * Significância estatística (p < 0,05).

## Discussão

Neste estudo, nós investigamos os efeitos dos padrões pressóricos dipper e não-dipper nos resultados laboratoriais e na qualidade de vida de pacientes com DPOC. De acordo com os achados do nosso estudo, os pacientes com DPOC e padrão pressórico não-dipper apresentaram valores mais altos de PCR e Aix, bem como menor qualidade de vida devido à DPOC. O aumento na prevalência do padrões pressórico não-dipper com o número de pessoas habitando o domicílio foi outro achado do estudo.

A MAPA 24h usada para diagnóstico e acompanhamento da hipertensão fornece informações sobre a presença de pressão dipper e não-dipper.^2^ Em nosso estudo, 76,1% dos pacientes com DPOC apresentaram o padrão pressórico não-dipper. Nersesyan et al. verificaram que a taxa do padrão pressórico não-dipper em pacientes com DPOC foi de 72,7%.^15^ No estudo realizado por Aidar et al.,^8^ o descenso na pressão noturna foi superior a 10%, em relação à pressão diurna, no grupo controle saudável, enquanto o valor médio da pressão noturna em pacientes com DPOC foi inferior a 10%, em relação à pressão diurna.^8^ Embora os dados de frequência não tenham sido compartilhados no estudo de Aidar et al.,^8^ entende-se que a queda da pressão arterial noturna era insuficiente em pacientes com DPOC. A presença de processos inflamatórios com base em ambas as condições pode explicar a associação entre a pressão não-dipper e a DPOC. No entanto, os efeitos de cada fator um sobre o outro ainda não são claros.^8^ Neste estudo, os níveis de PCR foram mais altos entre os indivíduos com padrão pressórico não-dipper. Um estudo anterior conduzido por Kaya et al.^11^ apresentou o mesmo achado, onde os valores de PCR foram mais altos em pacientes com padrão não-dipper, em comparação com pacientes com o padrão dipper.^11^ Estudos prévios têm demonstrado que a pressão não-dipper diminui o número de células progenitoras endoteliais, interrompe os mecanismos de reparo vascular e a homeostase endotelial e conseqüentemente induz a inflamação, aumentando a expressão de citocinas endoteliais.^5^ Em nosso estudo, sugerimos que os níveis mais elevados de PCR em indivíduos com padrão pressórico não-dipper foram causados por mecanismos semelhantes.

Em nosso estudo, não houve diferença significativa entre os valores de NLR e PLR com respeito aos padrões pressóricos. Estudos anteriores mostraram que os valores de NLR e PLR são mais elevados em pacientes com pressão arterial não-dipper.^5^ Golpe et al. investigaram a relação entre o padrão pressórico e marcadores inflamatórios em pacientes com DPOC, concluindo que o padrão pressórico não afeta os níveis de neutrófilos e linfócitos.^16^ Visto que a amostra do nosso estudo consistia apenas de pacientes com DPOC e que as vias inflamatórias desempenham um papel importante na DPOC, os valores de NLR e PLR podem não ter sido afetados pelo padrão pressórico.

Como resultado de nosso estudo, encontramos valores de Aix mais altos entre os pacientes com pressão arterial não-dipper; este achado está de acordo com os resultados de estudos anteriores na literatura.^4^ O Aix é considerado um dos melhores indicadores de rigidez arterial e condições relacionadas à aterosclerose e acredita-se que ele preveja eventos cardiovasculares próximos.^3^ A pressão arterial não-dipper desencadeia o processo inflamatório e causa aterosclerose e arteriosclerose, e acredita-se que esteja associada ao aumento do risco de mortalidade cardiovascular e danos aos órgãos-alvo.^2 , 3^ Considerando o risco aumentado de inflamação e aterosclerose causado pela pressão arterial não-dipper, pode-se esperar que o Aix seja mais alto em indivíduos com padrão pressóricos não-dipper. Como resultado desse achado corroborado por nosso estudo, pode ser útil monitorar de perto os pacientes com DPOC e o padrão pressórico não-dipper, a fim de melhorar o manejo de doenças cardiovasculares.

Em nosso estudo, a qualidade de vida geral medida pela EQ-5D foi menor em pacientes com padrões pressórico não-dipper de acordo com as análises univariadas; no entanto, essa relação perdeu sua importância nas análises multivariadas. As pontuações da escala de qualidade de vida do SGRQ, que são específicas para a DPOC, foram mais altas em pacientes com padrão pressórico não-dipper e essa relação permaneceu significativa nas análises multivariadas. Um dos motivos para isso pode ser o fato de que a EQ-5D mede a qualidade de vida geral, enquanto o SGRQ é específico para a DPOC. Considerando que a qualidade de vida geral de indivíduos com DPOC é inferior à de indivíduos saudáveis, o padrão pressórico não-dipper em pacientes com DPOC pode diminuir a qualidade de vida ainda mais.^17^ O estudo de Wacker et al. relatou que, em associação com a carga da doença, a pontuação do SGRQ prediz exacerbações agudas da DPOC, hospitalizações devido a exacerbações e mortalidade devido a todas as causas.^9^ Em indivíduos com o padrão pressórico não-dipper, a nossa pontuação média no SGRQ foi mais alto; isto pode indicar a importância do controle da pressão arterial durante o manejo da DPOC.

Em nosso estudo, verificamos que o aumento no número de pessoas habitando o domicílio aumentou a prevalência do padrão pressórico não-dipper. Maior número de pessoas morando no mesmo domicílio e o número de filhos são fatores que aumentam a responsabilidade e, consequentemente, levam ao estresse em indivíduos.^18^ Visto que o estresse fisiologicamente desencadeia a liberação da epinefrina e da norepinefrina, pode-se esperar que o padrão pressórico não-dipper seja mais frequente em pessoas com fatores de alto estresse.^19^ Além disso, a taxa de superlotação descreve a proporção de pessoas que vivem em moradias superlotadas, conforme definido pelo número de cômodos disponíveis para a família, o tamanho da família, a idade de seus membros e a sua situação familiar; 40% da população da Turquia vive em moradias superlotadas.^20^ Esta realidade pode constituir um alto risco para o a população turca em relação ao desenvolvimento do padrão pressórico não-dipper.

É necessário mencionar que existem algumas limitações em nosso estudo. Primeiro, considerando que o nosso estudo é transversal, há um problema de temporalidade em que não podemos ter certeza se o fator precedeu ou não a ocorrência do desfecho. Em nosso caso, não sabemos se a inflamação causou o padrão pressórico não-dipper ou vice-versa; portanto, estudos prospectivos são necessários para avaliar a causalidade. A não inclusão de um grupo de controle saudável pode ser uma limitação. Comparar indivíduos saudáveis com pacientes com DPOC pode fornecer melhores informações sobre os efeitos dos padrões pressóricos. Além disso, em nosso estudo, não foi investigada a presença de síndrome da apneia do sono. Tem sido verificado que o padrão pressórico não-dipper é alto em pacientes com síndrome da apnéia do sono e a síndrome da apnéia do sono é comum em pacientes com DPOC.^21 , 22^ A nossa taxa de participação foi menor do que a esperada; no entanto, determinamos diferenças nos principais indicadores, como PCR e as pontuações de qualidade de vida EQ5D e SGRQ, que estavam relacionados às hipóteses primárias e alcançamos mais de 90% nos cálculos de poder post hoc para esses parâmetros.

## Conclusão

O padrão pressórico não-dipper foi mais comum em pacientes com DPOC e os pacientes com DPOC e pressão arterial não-dipper apresentaram níveis de PCR e valores Aix mais elevados. Isto representa um risco aumentado de doenças cardiovasculares e lesões em órgãos-alvo. Ao mesmo tempo, o padrão não-dipper de pressão arterial afeta adversamente a qualidade de vida dos pacientes com DPOC e isso parece afetar negativamente as exacerbações da doença, hospitalizações e mortalidade. Por estes motivos, o monitoramento rigoroso da pressão arterial em pacientes com DPOC pode contribuir para o aumento da qualidade de vida individual e a redução dos níveis de mortalidade relacionados às doenças cardiovasculares.
