# Supplementary material for: Avaliação de Padrões Pressóricos Dipper e Não-Dipper e Qualidade de Vida entre Pacientes com Doença Pulmonar Obstrutiva Crônica
Source: Arq Bras Cardiol. 2021 Jan 13;116(2):295–302. [Article in Portuguese] doi: 10.36660/abc.20190536 (PMC7909972; doi:10.36660/abc.20190536)
Supplement: Translation

# Translation: Evaluation of Dipper and Non-dipper Blood Pressure Patterns and Quality of Life Among Patients with Chronic Obstructive Pulmonary Disease

**Authors:** Meryem Askın, Esra Meltem Koc, Kaan Sozmen, Muzaffer Onur Turan, Zeki Soypacacı, Saliha Aksun

**Affiliations:** 1 Turkey Izmir Katip Celebi University Faculty of Medicine - Department of Family Medicine, Izmir - Turkey; 2 Turkey Izmir Katip Celebi University Faculty of Medicine - Department of Public Health, Izmir - Turkey; 3 Turkey Izmir Katip Celebi University Faculty of Medicine - Department of Chest Diseases Izmir, - Turkey; 4 Turkey Izmir Katip Celebi University Faculty of Medicine - Department of Nephrology Izmir, - Turkey; 5 Turkey Izmir Katip Celebi University Faculty of Medicine - Department of Medical Biochemistry, Izmir - Turkey

**Keywords:** Pulmonary Disease Chronic Obstructive, Monitoring, Dipper, No-Dipper, Prognosis, Quality of Life

## Abstract

**Background:**

Non-dipper blood pressure is defined by less than a 10% reduction in nighttime blood pressure, and it is associated with cardiovascular disease. Inflammation is thought to play a role in the pathogenesis of both chronic obstructive pulmonary disease (COPD) and non-dipper blood pressure pattern, and both diseases are associated with lower quality of life.

**Objective:**

The aim of this study was to investigate the effects of non-dipper blood pressure pattern in patients with COPD.

**Methods:**

A cross-sectional study was carried out with 142 patients with COPD. The Saint George Respiratory Questionnaire and the Euro Quality of Life Scale were used to collect data. To understand arterial stiffness, the augmentation index and pulse wave velocity were measured, and 24-hour ambulatory blood pressure monitoring was subsequently performed. A multivariable logistic regression model was used to understand the relationship between different independent variables and blood pressure pattern. P values lower than 0.05 were considered statistically significant.

**Results:**

As a result, 76.1% (n = 108) of the patients had non-dipper blood pressure pattern. Non-dipper patients had higher C-reactive protein (OR:1.123; 95% CI:1.016;1.242), augmentation index (OR: 1.057; 95% CI: 1.011;1.105) and Saint George Respiratory Questionnaire total score (OR: 1.021; 95% CI: 1.001;1.042) than dipper patients. Also, as the number of people living at home increased, non-dipper blood pressure pattern was found to be more frequent (OR: 1.339; 95% CI: 1.009;1.777).

**Conclusion:**

Non-dipper blood pressure pattern may increase cardiovascular risk by triggering inflammation and may adversely affect the prognosis of COPD by lowering the disease-related quality of life. (Arq Bras Cardiol. 2020; [online].ahead print, PP.0-0)

## Introduction

Chronic obstructive pulmonary disease (COPD) is highly prevalent worldwide; it is characterized by the limitation of airway flow and, according to the World Health Organization, it will be the third leading cause of death in 2030.^1^

A reduction in blood pressure by more than 10% at night is a physiological process^2^. If the reduction in blood pressure at night is less than 10%, it is called the non-dipper blood pressure pattern, which is known to be associated with cardiovascular disease and end-organ damage.^2^The augmentation index (Aix) and pulse wave velocity (PWV) are indicators of arterial stiffness that can predict future cardiovascular events, and Aix and PWV values are higher in the non-dipper blood pressure pattern.^3 , 4^It is controversial how non-dipper blood pressure pattern causes these effects, but it is thought that this pattern induces cytokine expression from the endothelium and triggers an inflammatory process.^5^Inflammation markers such as C-reactive protein (CRP), the neutrophil-lymphocyte ratio (NLR), and the platelet-lymphocyte ratio (PLR) are higher among individuals with non-dipper blood pressure compared to their counterparts.^5^

Systemic inflammation plays a major role in the pathogenesis of COPD, and it is believed that CRP and NLR can be used as markers for diagnosis and prognosis of COPD.^6 , 7^The presence of an inflammatory process in the pathogenesis of both COPD and non-dipper blood pressure suggests that the incidence of non-dipper blood pressure pattern may be high in patients with COPD.^8^Another common point of both diseases is their negative effects on quality of life.^9 , 10^Quality of life is the health perception of the individual, and it has a direct impact on physical and mental health; it is also related to the number of acute exacerbations and hospitalizations in COPD.^9^

Studies that have investigated the effects of blood pressure patterns on COPD are quite limited, and elucidation of this subject might be a guide for COPD management. The aim of this study is to investigate the effect of non-dipper blood pressure pattern on laboratory values and quality of life in COPD.

## Methods

The diagnosis of COPD should be confirmed via spirometry in patients with dyspnea, chronic cough, chronic sputum production, and exposure to risk factors of COPD. In our study, patients diagnosed with COPD by chest disease specialists, who were therefore taking medication, were accepted as COPD. In addition, spirometry examinations of patients were checked by the computer system.

### Participants

This cross-sectional study was conducted with patients with COPD, between the ages of 18 and 80 years, who were admitted to Izmir Katip Celebi University Atatürk Training and Research Hospital’s inpatient or outpatient clinic for pulmonary diseases. The study excluded patients who, in addition to COPD, had known active infection, malignancy, congestive heart failure, diabetes mellitus, or renal failure. The study was conducted between January and June 2018.

### Sample size

The sample size was calculated using the GPOWER 3.1 program. By using a previous research finding, we assumed the mean CRP levels would be 4.9 ± 1.7 mg/lt in the non-dipper group and 3.8 ± 1.5 mg/lt in the dipper group. In order to show the differences between the two groups, the required sample size was calculated at 128, with 95% power and a two-sided type-1 error rate of 5%. We increased the sample size by 10% and aimed to reach 140 patients.^11^

### Data collection tools

#### Sociodemographic characteristics

In order to examine sociodemographic data, education level was categorized into two groups (middle school or lower and high school or higher). Income level was categorized into three groups (≥ 1500 Turkish Liras (TL), 1501 to 3499 TL, and ≤ 3500 TL). Body mass index values were divided into three categories (normal [≤ 24.99 kg/m^2^], overweight [25 to 29.99 kg/m^2^], and obese [≥ 30 kg/m^2^]). Marital status was divided into two groups (single and married). Alcohol intake was also divided into two groups (yes and no). Places where patients were included in the study were divided into two groups (inpatient service and outpatient clinic), and COPD history in their families was categorized into two groups (yes and no). Patients diagnosed with hypertension by a physician and receiving prescription medication were considered as having hypertension. Patients who smoked regularly, at least once a day, were considered as active smokers. Regarding continuous variables, patients were asked to respond to questions such as age, number of people living at home, years of smoking and package numbers, number of admissions to the emergency unit due to COPD during the last year, and number of hospital admissions for COPD.

#### Saint George’s Respiratory Questionnaire

Saint George’s Respiratory Questionnaire (SGRQ) is a quality of life scale especially developed for the respiratory system that can be used in patients with asthma and COPD, as well as in patients with bronchiectasis and sarcoidosis.^12^The scale includes the components of symptoms, activity, and effect, and, after scoring, all three components and total quality of life scores are obtained.^12^The scale is scored from 0 to 100. For each component and for the produced total score, ‘0’ indicates ‘perfect’ and ‘100’ indicates ‘worst’ quality of life.^12^The validity and reliability of the Turkish version of the scale have been confirmed.^12^

#### Euro Quality of Life Scale

The Euro Quality of Life Scale (EQ-5D) is a 5-question overall quality of life scale, each consisting of 3 levels.^13^The scale is composed of 5 dimensions, including mobility, self-care, daily activities, pain, and mood. Higher scores indicate higher quality of life.^13^The utility of the EQ-5D score was computed using the MVH-A1 algorithm by Dolan.^14^This algorithm provides a range from −0.594 to +1, where higher values indicate better quality of life. The scale’s validity and reliability have been confirmed in Turkish settings.^13^

#### Augmentation index and pulse wave velocity

After the administration of the questionnaire and blood sampling, patients were examined in terms of Aix and PWV. A Mobil-O-Graph® (IEM; Stolberg, Germany) device calculated the Aix and PWV by recording oscillometric brachial blood pressure; the cuff subsequently reinflated at the diastolic phase for approximately 10 seconds, recording brachial pulse waves with a high-fidelity pressure sensor.

#### 24-hour ambulatory blood pressure monitoring and blood sampling

For 24-hour blood pressure measurement, patients were monitored with a Mobil-O-Graph NG® (IEM; Stolberg, Germany) device. The device was adjusted to perform a 24-hour measurement, once every 15 minutes during the day and once every 30 minutes at night. The next day, at the same time, patient returned the devices. The non-dominant arm was used for measurement, and day and night measurements were adjusted according to the patients’ sleeping and waking hours. Patients were instructed to continue their usual activities and to avoid exhausting exercise. Blood tests were obtained from patients before treatment was administered. Among patients who were hospitalized in the pulmonary diseases service, the questionnaire was applied on the same day. The 24-hour ambulatory blood pressure monitoring (24-h ABPM) measuring device was applied in similar conditions for outpatients.

The results of 24-h ABPM measurement were examined, and, if nighttime average values of systolic and diastolic blood pressures were decreased by 10% or more with respect to day-time average values, this was considered as dipper blood pressure^2^. When the decrease in blood pressure was less than 10%, it was considered as non-dipper blood pressure pattern.^2^CRP blood tests were carried out in a Architect C16000 autoanalyzer (Abbott Diag., USA), and the hemogram was analysed in a Mindray BC-6800 whole blood device (Mindray, China).

#### Ethical approval

The ethical approval for the study was obtained from the Izmir Katip Celebi University Interventional Clinical Research Ethics Committee under decision number 164 (date of approval: December 22, 2016). All the procedures in this study were in accordance with the 1975 Helsinki Declaration, updated in 2013. Informed consent was obtained from all participants included in the study.

#### Statistical analysis

Statistical analysis was performed using SPSS version 16. In this study, the distribution of the continuous variables were assessed with Kolmogorov-Smirnov test, and the data were not distributed normally. Continuous variables were presented as medians and inter quartile ranges (IQR). Statistical comparison of two independent groups was performed using the Mann Whitney U test. The between-group comparisons of categorical variables were performed using the chi-square test. The strength of association between two continuous variables was assessed using Spearman correlation tests.

Independent effects with respect to the presence of non-dipper blood pressure of the different identifying factors were examined using logistic regression models. The Hosmer-Lemeshow test was used for assesing the model fit. Independent variables with p ≤ 0.10 on bivariate analysis were included in the multivariate logistic regression model with a “backward” elimination method. Due to high correlation between nighttime systolic blood pressure and nighttime mean arterial pressure, only nighttime mean arterial pressure was added to the model. Adjusted coefficients are presented with their 95% confidence intervals. All p values were two-tailed, and p values lower than 0.05 were considered statistically significant.

## Results

One hundred and sixty-seven patients were invited to the study. Twelve patients did not want to wear the 24-h ABPM device or did not want to participate in the study due to lack of time. Thirteen patients were excluded from the study due to inappropriate 24-h ABPM measurements, and the study was completed with 142 patients. In total, 23.9% (n = 34) of the patients had dipper and 76.1% (n = 108) had non-dipper blood pressure pattern. Among these patients, 43% (n = 61) participated from the inpatient clinic, and 57% (n = 81) were outpatients.

According to univariate analysis, sociodemographic variables such as age, sex, and marital status did not significantly affect the blood pressure pattern. On the other hand, the median number of people living in the household were higher in those who had non-dipper blood pressure pattern compared to those with dipper blood pressure pattern. The relationship between sociodemographic variables and blood pressure pattern is presented in Table 1 .

**Table 1:** – Sociodemographic variables

Categorical variables	Dipper	Non-dipper	Total	P value
	**(n = 34)**	**(n = 108)**	**(n = 142)**	
**Sex n (%)**				
Female	7 (20.6)	25(23.1)	32 (22.5)	0.819
Male	27 (79.4)	83 (76.9)	110 (77.5)	
**Body mass index n (%)**				
Normal (18.5 to 24.5)	19 (55.9)	51 (47.2)	70 (49.3)	0.571
Overweight (25 to 29,9)	10 (29.4)	33 (30.6)	43 (30.3)	
Obese (30 to 40)	5 (14.7)	24 (22.2)	29 (20.4)	
**Level of education n (%)**				
Middle school and lower	11 (32.4)	43 (39.8)	54(38)	0.544
High school and higher	23(67.6)	65 (60.2)	88 (62)	
**Income level n (%)**				
1500 Turkish liras or below (low)	0 (0)	9 (8.3)	9 (6.3)	0.217
1501 to 3500 Turkish lira (medium)	20 (58.8)	60 (55.6)	80 (56.3)	
3501 Turkish liras or higher (high)	14 (41.2)	39 (36.1)	53 (37.3)	
**Marital status n (%)**				
Single	9 (26.5)	24 (22.2)	33 (23.2)	0.644
Married	25 (73.5)	84 (77.8)	109 (76.8)	
**Place of attendance n (%)**				
Inpatient services	11 (32.4)	50 (46.3)	61 (43)	0.169
Outpatient clinic	23 (67.6)	58 (53.7)	81 (57)	
**Presence of hypertension n (%)**				
Yes	18 (52.9)	49 (47.1)	67 (48.6)	0.693
No	16 (47.1)	55 (52.9)	71 (51.4)	
**Family history of COPD n (%)**				
Yes	9 (26.5)	43 (39.8)	52 (36.6)	0.221
No	25 (73.5)	65 (60.2)	90 (63.4)	
**Smoking n (%)**				
Yes	8 (23.5)	21 (29.4)	29 (20.4)	0.629
No	26 (76.5)	87 (80.6)	113 (79.6)	
**Use of alcohol n (%)**				
Yes	12 (35.3)	25 (23.1)	37 (26.1)	0.182
No	22 (64.7)	83 (76.9)	105 (73.9)	
**Non-categorical variables**				
Age, median (IQR)	65 (59.75-73)	66 (55-71.75)	66 (55.75-72)	0.739
Number of people living at home, median (IQR)	2 (1-2)	2 (2-3)	2 (2-3)	0.038^*^
Smoking, pack years, median (IQR)	42.5 (23.75-60)	50 (30-80)	50 (30-74.25)	0.317
Number of visits to emergency department in the last year, median (IQR)	3,5 (1-6)	3 (1.25-6)	3 (1-6)	0.929
Number of hospitalization in the last year, median (IQR)	2 (0-3)	1 (0-2)	1 (0-2)	0.296

p: percentiles; IQR: interquartile range. * Statistical significance (p < 0.05)

Mean nighttime systolic blood pressure, mean nighttime arterial pressure, Aix, and CRP values were significantly higher among participants with non-dipper blood pressure pattern compared to thier counterparts. The association between 24-hour blood pressure and laboratory results are shown in Table 2 . When patient quality of life was examined according to blood pressure pattern, SGRQ symptoms, effects, and total scores were higher among the patients with non-dipper blood pressure, and the EQ-5D score was lower; the differences were statistically significant. ( Table 3 ).

**Table 2:** – 24-Hour Ambulatory Blood Pressure Values and Laboratory

Blood pressure values and laboratory results	Dipper (n = 34)	Non-dipper (n = 108)	Total (n = 142)	P value
24-h mean SBP, mmHg, median (IQR)	122 (111-129)	122.5 (116-129.75)	122 (115.75-129.25)	0.985
24-h mean DBP, mmHg, median (IQR)	76 (71- 85.75)	73.5 (67.25-82)	74 (68-84)	0.236
24-h mean AP, mmHg, median (IQR)	97 (88-113)	97 (90-105)	97 (88.75-106)	0.305
24-h mean heart rate, median (IQR)	84 (69.75-88)	77 (70-84.75)	77.5 (70-88)	0.152
24-h pulse pressure, mmHg, median (IQR)	47 (37-51)	47 (42-52)	47 (42-51.25)	0.979
Daytime mean SBP, mmHg, median (IQR)	128 (110.75-145)	124 (116-131)	124.5 (116-133)	0.224
Daytime mean DBP, mmHg, median (IQR)	74 (71-83)	75 (67-82)	74 (69-82)	0.622
Daytime mean AP, mmHg, median (IQR)	99 (73.25-91.5)	97 (90-104)	98 (91-106)	0.098
Daytime mean heart rate, median (IQR)	84 (73.25-91.5)	78 (71-86.75)	80 (71-87)	0.127
Daytime pulse pressure, mmHg, median (IQR)	47 (38-52.25)	47 (42-52)	47 (42-52)	0.781
Nighttime mean SBP, mmHg, median (IQR)	118 (107.75-120)	120 (115-133)	118 (114-131.5)	0.012^*^
Nighttime mean DBP, mmHg, median (IQR)	71 (64.75-73)	72 (65-80)	72 (65-78)	0.099
Nighttime mean AP, mmHg, median (IQR)	94 (82.5-94)	94 (87-104.5)	94 (86-101)	0.045^*^
Nighttime mean heart rate, median (IQR)	74.5 (54-88)	73 (61-82)	73 (61-82)	0.698
Nighttime pulse pressure, mmHg, median (IQR)	44 (43.25-51.5)	47.5 (44-52)	47 (44-52)	0.105
Augmentation index, median (IQR)	20.40 (14.75-24.3)	27 (18-35)	23.05 (17-33)	0.020^*^
Pulse wave velocity, median (IQR)	9.65 (8.67-10.32)	9.2 (8-10.2)	9.45 (8.2-10.8)	0.794
WBC, median (IQR)	8.85 (7.71-10.63)	9.61 (8.64-11.18)	9.44 (8.32-11.14)	0.073
CRP, median (IQR)	0.97 (0.02-3)	4.34 (1.12-7.42)	2.86 (0.6-6.54)	<0.001^*^
NLR, median (IQR)	2.89 (2.29-5.47)	3.93 (2.28-6.19)	3.76 (2.31-5.88)	0.210
PLR, median (IQR)	130.5 (95.16-203.58)	160.37 (115.38-201.61)	152.06 (107.74-201)	0.110

SBP: Systolic blood pressure; DBP: Diastolic blood pressure; AP: arterial pressure; WBC: White blood cells; CRP: C reactive protein; NLR: neutrophil lymphocyte ratio; PLR: platelet lymphocyte ratio; IQR: interquartile range. * Statistical significance (p <0.05)

**Table 3:** – Saint George’s Respiratory Questionnaire and Euro Quality of Life Scale scores

Scores	Dipper (n = 34)	Non-dipper (n =108)	Total (n = 142)	p value
SGRQ Symptoms, median (IQR)	74.13 (56.43-78.97)	78.08 (64.97-83.68)	76.08 (61.4-82.27)	0.021^*^
SGRQ Activity, median (IQR)	62.49 (48.38-86.72)	74.48 (60.67-87.53)	71.4 (53.83-87.53)	0.091
SGRQ Impact, median (IQR)	47.64 (25.15-67.32)	58.1 (46.33-74.77)	56.99 (38.33-74.3)	0.025^*^
SGRQ Total, median (IQR)	53.84 (35.64-73.64)	65.23 (53.84-79.85)	63.71 (52.01-78.63)	0.021^*^
EQ-5D, median (IQR)	0.51 (0.51-0.58)	0.51 (0.51-0.51)	0.51 (0.51-0.51)	0.034^*^

p: percentiles; SGRQ : Saint George’s Respiratory Questionnaire; EQ-5D: Euro Quality of Life Scale; IQR: interquartile range. * Statistical significance (p < 0.05)

According to multivariate logistic regression analysis, CRP (OR: 1.123; 95% CI: 1.016;1.242), Aix (OR: 1.057; 95% CI: 1.011;1.105), and SGRQ total score (OR: 1.021; 95% CI: 1.001;1.042) were higher in patients with non-dipper blood pressure pattern than in those with dipper blood pressure. In addition, individuals with non-dipper blood pressure pattern were living in more crowded houses, compared to individuals with dipper blood pressure pattern (OR: 1.339; 95% CI: 1.009;1.777) ( Table 4 ).

**Table 4:** – Logistic regression analysis of non-dipper blood pressure

Determining factors	Odds ratio (Univariate)			Odds ratio (Multivariate)		
	β	95% CI	p value	β	95% CI	p value
Age	0.997	0.961;1.034	0.879	0.995	0.950;1.042	0.817
**Sex**						
Female	1	1	1	1	1	1
Male	0.861	0.335;2.212	0.756	1.100	0.341;3.546	0.873
Number of people living at home	1.169	0.903;1.515	0.236	1.339	1.009;1.777	0.043*
Nighttime mean AP, mmHg	1.038	0.999;1.078	0.055	1.033	0.991;1.077	0.122
Augmentation index	1.049	1.008;1.092	0.018*	1.057	1.011;1.105	0.015**
CRP	1.141	1.025;1.270	0.016*	1.123	1.016;1.242	0.024*
SGRQ Total	1.023	1.003;1.043	0.025*	1.021	1.001;1.042	0.040*
EQ-5D	0.440	0.076;2.540	0.358	0.766	0.118;4.960	0.780

β: regression coefficient; 95% CI: confidence interval; AP: arterial pressure; CRP: C reactive protein; SGRQ : Saint George’s Respiratory Questionnaire; EQ-5D: Euro Quality of Life Scale. * Statistical significance (p < 0.05)

## Discussion

In this study, we investigated the effects of the dipper and non-dipper blood pressure patterns on laboratory results and quality of life of patients with COPD. According to our study findings, patients with COPD and non-dipper blood pressure pattern had higher CRP and Aix values and lower quality of life due to COPD. The increase in the prevalence of the non-dipper blood pressure pattern with the number of people living at home is another finding of the study.

The 24-h ABPM used for diagnosis and follow-up of hypertension provides information about the presence of dipper and non-dipper blood pressure.^2^In our study, 76.1% of patients with COPD had a non-dipper blood pressure pattern. Nersesyan et al. found that the rate of non-dipper blood pressure pattern in patients with COPD was 72.7%.^15^In the study performed by Aidar et al.,^8^the decrease in nighttime blood pressure was higher than 10%, with respect to daytime blood pressure, in the healthy control group, while the mean value of nighttime blood pressure in patients with COPD was below 10%, with respect to daytime blood pressure.^8^Although the frequency data were not shared in the study by Aidar et al.,^8^it was understood that the decrease in nighttime blood pressure of patients with COPD was insufficient. The presence of inflammatory processes based on both conditions might explain the association between non-dipper blood pressure and COPD. However, the effects of each factor on one another are still unclear.^8^In this study, CRP levels were higher among individuals with non-dipper blood pressure pattern. A previous study conducted by Kaya et al.^11^presented the same finding, where CRP values were higher in patients with non-dipper pattern, compared to patients with dipper blood pressure.^11^Previous studies have shown that non-dipper blood pressure decreases the number of endothelial progenitor cells, disrupts vascular repair mechanisms and endothelial homeostasis, and consequently induces inflammation by increasing the expression of endothelial cytokines.^5^In our study, we suggest that the higher CRP levels in individuals with the non-dipper blood pressure pattern were caused by similar mechanisms.

In our study, there was no significant difference between NLR and PLR values based on blood pressure patterns. Previous studies have shown that NLR and PLR values are higher in patients with non-dipper blood pressure.^5^Golpe et al. investigated the relationship between blood pressure pattern and inflammatory markers in patients with COPD, and they concluded that blood pressure pattern does not affect neutrophil and lymphocyte levels.^16^Given that the sample of our study only consisted of patients with COPD and that inflammatory pathways play a major role in COPD, the NLR and PLR values may not have been affected by the blood pressure pattern.

As a result of our study, we found higher Aix values among patients with non-dipper blood pressure; this finding is in concordance with the results of previous studies in the literature.^4^Aix is considered as one of the best indicators of arterial stiffness and atherosclerosis-related conditions, and it is thought to predict oncoming cardiovascular events.^3^Non-dipper blood pressure triggers the inflammatory process and causes atherosclerosis and arteriosclerosis, and it is thought to be associated with increased risk for both cardiovascular mortality and end-organ damage.^2 , 3^Considering the increased risk of inflammation and atherosclerosis caused by non-dipper blood pressure, Aix might be expected to be higher in individuals with non-dipper blood pressure pattern. As a result of this finding supported by our study, it might be useful to closely monitor patients with COPD and non-dipper blood pressure pattern, in order to improve management of cardiovascular diseases.

In our study, the general quality of life measured by EQ-5D was lower in patients with non-dipper blood pressure pattern according to univariate analyses; however, this relationship lost its significance in multivariate analyses. SGRQ quality of life scale scores, which were specific for COPD, were higher in patients with non-dipper blood pressure pattern, and this relationship remained significant in multivariate analyses. One reason for this could be due to the fact that the EQ-5D measures the overall quality of life, while the SGRQ is specific for COPD. Considering that the overall quality of life of individuals with COPD is lower than that of healthy individuals, the non-dipper blood pressure pattern in patients with COPD may further decrease the quality of life.^17^The study of Wacker et al. reported that, in association with disease burden, SGRQ score predicts acute COPD exacerbations, hospitalizations due to exacerbations, and deaths due to all causes.^9^In individuals with a non-dipper blood pressure pattern, our mean SGRQ score was higher, which may indicate the importance of blood pressure control during COPD management.

In our study, we found that the increase in the number of people living at home increased the prevalence of the non-dipper blood pressure pattern. Increased number of people living in the same house and the number of children are factors that increase the responsibility and hence lead to stress on individuals.^18^As stress physiologically triggers the release of epinephrine and norepinephrine, the non-dipper blood pressure pattern can be expected to be more frequent in people with high stress factors.^19^Moreover, the overcrowding rate describes the proportion of people living in overcrowded dwellings, as defined by the number of rooms available to the household, the household’s size, its members’ ages, and their family situation; 40% of the population of Turkey lives in overcrowded families.^20^This case may constitute a high risk for people of Turkey for the development of non-dipper blood pressure pattern.

It is necessary to mention that there are some limitations to our study. First, as our study is cross-sectional, there is a temporality problem where we cannot be sure whether the factor preceded the occurrence of the outcome or not. In our case, we do not know whether inflammation caused non-dipper blood pressure pattern or vice verse; hence, prospective studies are needed in order to assess causality. Failure to include a healthy control group may be a limitation. Comparisons of healthy individuals and patients with COPD might provide better information regarding the effects of blood pressure patterns. Also, in our study, the presence of sleep apnea syndrome was not investigated. Non-dipper blood pressure pattern was found to be high in patients with sleep apnea syndrome, and sleep apnea syndrome is common in patients with COPD.^21 , 22^Our participation rate was lower than expected; however, we determined differences in major indicators such as CRP and EQ-5D and SGRQ quality of life scores, which were related to the primary hypotheses, and we achieved more than 90% power in post hoc power calculations for these parameters.

## Conclusion

The non-dipper blood pressure pattern was more common in patients with COPD, and patients with COPD and non-dipper blood pressure had higher CRP levels and Aix values. This poses an increased risk of cardiovascular disease and end-organ damage. At the same time, the non-dipper blood pressure pattern adversely affects quality of life of patients with COPD, and this is thought to negatively affect exacerbations of the disease, hospitalizations, and mortality. For these reasons, close monitoring of blood pressure in patients with COPD may contribute to increased individual quality of life and decreased levels of mortality related to cardiovascular diseases.
